# Obstacle-Avoidance Planning in C-Space for Continuum Manipulator Based on IRRT-Connect

**DOI:** 10.3390/s25103081

**Published:** 2025-05-13

**Authors:** Yexing Lang, Jiaxin Liu, Quan Xiao, Jianeng Tang, Yuanke Chen, Songyi Dian

**Affiliations:** 1 State Grid Electric Power Research Institute of Liaoning Electric Power Co., Ltd., Shenyang 110000, China; bianyushan_ever@163.com (Y.L.); liujxldk@163.com (J.L.); jialuntang@126.com (J.T.); 2 College of Electrical Engineering, Sichuan University, Chengdu 610065, China; chenyuanke@stu.scu.edu.cn (Y.C.); scudiansy@scu.edu.cn (S.D.)

**Keywords:** continuum manipulator, unstructured environments, bidirectional rapidly exploring random tree, coppeliasim, path planning

## Abstract

Aiming at the challenge of trajectory planning for a continuum manipulator in the confined spaces of gas-insulated switchgear (GIS) chambers during intelligent operation and maintenance of power equipment, this paper proposes a configuration space (C-space) obstacle-avoidance planning method based on an improved RRT-Connect algorithm. By constructing a virtual joint-space obstacle map, the collision-avoidance problem in Cartesian space is transformed into a joint-space path search problem, significantly reducing the computational burden of frequent inverse kinematics solutions inherent in traditional methods. Compared to the RRT-Connect algorithm, improvements in node expansion strategies and greedy optimization mechanisms effectively minimize redundant nodes and enhance path generation efficiency: the number of iterations is reduced by 68% and convergence speed is improved by 35%. Combined with polynomial-driven trajectory planning, the method successfully resolves and smoothens driving cable length variations, achieving efficient and stable control for both the end-effector and arm configuration of a dual-segment continuum manipulator. Simulation and experimental results demonstrate that the proposed algorithm rapidly generates collision-free arm configuration trajectories with high trajectory coincidence in typical GIS chamber scenarios, verifying its comprehensive advantages in real-time performance, safety, and motion smoothness. This work provides theoretical support for the application of continuum manipulator in precision operation and maintenance of power equipment.

## 1. Introduction

With the continuous growth of electrical loads and consumer populations, the power industry has entered a phase of rapid development, accompanied by extensive deployment of generation, transmission, transformation, distribution, and consumption infrastructure. Consequently, the operation, maintenance, and inspection of critical power equipment have become pivotal in ensuring grid stability [1,2].

In confined maintenance environments such as gas-insulated switchgear (GIS), traditional rigid manipulators prove ill-suited for compact configurations due to their mechanical constraints. In contrast, cable-driven continuum manipulators demonstrate exceptional suitability for such inspection scenarios by leveraging their slender profile, redundant degrees of freedom, and enhanced adaptability [3].

Similar to rigid manipulators, investigating methods to minimize temporal costs in path planning and motion expenditure remains critically significant for inspection and maintenance tasks involving continuum manipulators. However, while the redundant degrees of freedom inherent to continuum manipulators enhance their maneuverability in confined environments, they concurrently increase the complexity of motion planning problems [4,5]. This necessitates the development of a robust trajectory planning methodology that explicitly accounts for the structural characteristics and kinematic constraints of continuum manipulators.

For conventional rigid manipulators, extensive research exists on path planning solutions employing genetic algorithms [6], ant colony optimization [7], and artificial potential field methods [8]. However, the redundancy characteristics of continuum manipulators have predominantly focused current investigations on imitation learning [9], optimization-based approaches [10], and reinforcement learning techniques [11]. Recent advancements in sampling-based planners have also introduced improved probabilistic roadmap (PRM) variants for complex multi-agent systems. For instance, Ref. [12] proposed a hierarchical PRM framework for multi-UAV path planning in cluttered 3D environments, demonstrating enhanced computational efficiency through obstacle decomposition, while Ref. [13] developed a congestion-aware multi-robot cooperative PRM optimizer to balance coverage and collision risks in dynamic settings. Despite these innovations, attempts to adapt classical PRM [14] or rapidly exploring random tree (RRT) [15] methods to continuum manipulators (e.g., in minimally invasive surgical robots [16,17]) still suffer from excessive redundant nodes that induce body distortion phenomena during deployment.

Most existing methods primarily conduct planning in Cartesian space with weak interaction between joint configurations and the environment. In recent years, some scholars have chosen to map obstacles from Cartesian space to the configuration space (C-space) [18], thereby providing more efficient solutions for tasks requiring arm-shape collision avoidance, eliminating extensive inverse kinematics computations and improving solving efficiency. However, due to the lack of effective mapping between the configuration space of cable-driven continuum manipulator and obstacle maps, planning efficiency cannot meet operational maintenance requirements.

In summary, this paper focuses on cable-driven continuum manipulators and proposes a C-space obstacle-avoidance trajectory planning method suitable for multi-segment continuum manipulators. The method addresses rapid shape collision detection, combines step-by-step optimization and greedy strategies to effectively improve solving efficiency and reduce node count, and further optimizes and smooths the driving trajectory. By employing MATLAB, CoppeliaSim Edu V4.2.0, and physical hardware co-simulation, both simulations and experiments demonstrate the algorithm’s rapidity and the accuracy of arm-shape planning.

## 2. Machine Design

**Figure 1:**
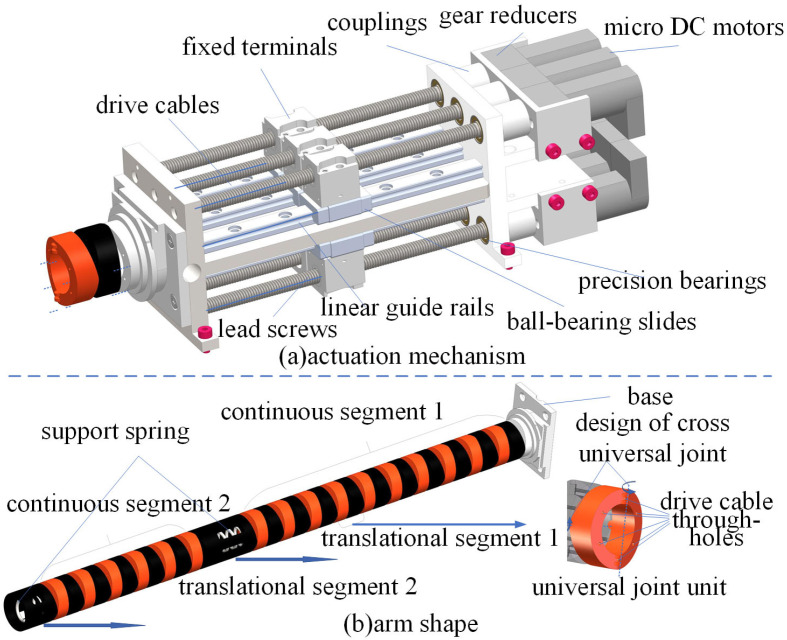
Structural diagram of continuum manipulator.

Currently, existing actuation methods for continuum manipulators include individual cable/tendon-driven systems, shape memory alloy actuation, pneumatic artificial muscle actuation, and hybrid actuation based on electroactive reactants and fluids. Due to the narrow and complex internal environment of GIS chambers, the designed actuation mechanism requires miniaturization to enable the manipulator to better perform various tasks. Compared to other solutions, the cable-driven approach utilizes micromotors to control manipulator motion, which meets miniaturization design requirements, thus selecting cable-driven actuation for continuum manipulators. The designed actuation mechanism, as shown in Figure 1, consists of micro DC motors, gear reducers, precision bearings, couplings, ball-bearing slides, linear guide rails, lead screws, drive cables, and fixed terminals. The actuation mechanism has dimensions of 140 mm × 50 mm × 42 mm and can be directly mounted onto the mobile platform of GIS chamber maintenance robots.

The continuum body, as the core structure of the manipulator, must be designed to ensure motion in complex environments through bending and rotation. Inspired by the universal joint principle, a continuum manipulator body structure incorporating universal joint units is designed, as shown in Figure 1. This structure features a hollow cylindrical design with an outer diameter of 20 mm, inner diameter of 10 mm, and total length of 304 mm, with an internal spring installed for support. The continuum body comprises two continuous segments and two translational segments. Each continuous segment consists of multiple sets of universal joint units connected in series, enabling bending and rotational motion, while the translational segments are used for mounting actuators or securing drive cables. Drive cable through-holes are reserved on the circumferential cross-section of the continuum body.

## 3. Problem Description and Kinematics Analysis

### 3.1. Problem Description

Continuum manipulator motion planning essentially involves generating continuous actuator trajectories from a starting point to a target point within static or dynamic workspaces; these actuator trajectories are typically continuous functions of time.

The entire configuration space C is composed of the free configuration space $C_{free}$ and the obstacle configuration space $C_{obs}$. Given the initial virtual joint configuration $q_{init}\left(q_{init}\in C_{free}\right)$ and the target virtual joint configuration $q_{goal}\left(q_{goal}\in C_{free}\right)$ of continuum manipulator 𝒜, the path planning aims to find a configuration sequence Q from $q_{init}$ to $q_{goal}$ that satisfies kinematic collision-free constraints, i.e.,

(1) $$Q=\left(q_{0},q_{1},q_{2}\ldots ,q_{i}\right),i=0,1,2,\ldots ,k\;\begin{array}{c} s.t.\begin{array}{l} q_{0}=q_{init},q_{k}=q_{goal} \end{array}\\ q_{i}\in C_{free} \end{array}$$

The point-to-point motion planning problem for continuum manipulators can be described as follows. In complex GIS environments, given the initial configuration $q_{init}$ and target configuration $q_{goal}$ of the continuum manipulator, a continuous function is obtained mapping $l:[0,t_{f}]\to L$ for the drive cable trajectory with respect to time t, such that the joint configuration satisfies $q\left(L\left(0\right)\right)=q_{init},q\left(L\left(t_{f}\right)\right)=q_{goal}$, and for any time $t\in [0,t_{f}]$, $q\left(L\left(t\right)\right)\in C_{free}$. The virtual joint configuration $q\left(L\left(t\right)\right)$ at any time t is derived from the two-segment continuum manipulator’s forward kinematics relationship $FK_{1}$.

**Figure 2:**
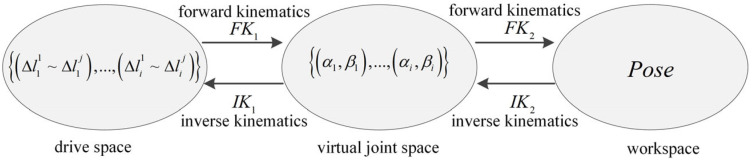
Kinematic mapping relationships of continuum manipulator.

As shown in Figure 2, after obtaining the obstacle-avoidance configuration, the inverse kinematics (IK) from the virtual joint space to drive space is utilized to derive the drive cable variation time sequence $L\left(t\right)$, i.e.,

(2) $$L\left(t\right)=\left\{L\left(q^{t_{0}}\right),\ldots ,L\left(q^{t_{1}}\right),\ldots ,L\left(q^{t_{f}}\right)\right\}$$

where $L\left(q^{t_{i}}\right)=\left(\Delta l_{1}^{1}\left(q^{t_{i}}\right),\;\Delta l_{1}^{2}\left(q^{t_{i}}\right),\;\Delta l_{1}^{3}\left(q^{t_{i}}\right),\;\Delta l_{2}^{1}\left(q^{t_{i}}\right),\;\Delta l_{2}^{2}\left(q^{t_{i}}\right),\;\Delta l_{2}^{3}\left(q^{t_{i}}\right)\right)$ represents the variation amounts of six driven cables from their original lengths at the corresponding time instances. Here, $q^{t_{i}}$ denotes the joint configuration $q_{i}$ reached at time $t_{i}$.

### 3.2. Kinematics Analysis of Continuum Manipulators

For the cable-driven continuum manipulator, a geometric model based on the PCC (Piecewise Constant Curvature) assumption is established, introducing virtual joint-space variables $\left(\alpha ,\beta \right)$ to represent the rotation angle and bending angle of a single segment. Under this framework, the kinematic model of the cable-driven continuum manipulator comprises two parts: the mutual mapping between the drive space (composed of all drive cables) and the artificially introduced virtual joint space, and the mutual mapping between the virtual joint space and the manipulator’s end-effector workspace. The forward kinematics relationships FK include the mapping from the drive space to virtual joint space $FK_{1}$ and from the virtual joint space to workspace $FK_{2}$. The inverse kinematics relationships IK consist of the mapping from the workspace to virtual joint space $IK_{2}$ and from the virtual joint space to drive space $IK_{1}$. As shown in Figure 2, the subscripts i in the first two spaces denote the i-th continuum segment, while the superscript j indicates the number of drive cables required for that segment, which can be 3 or 4.

**Figure 3:**
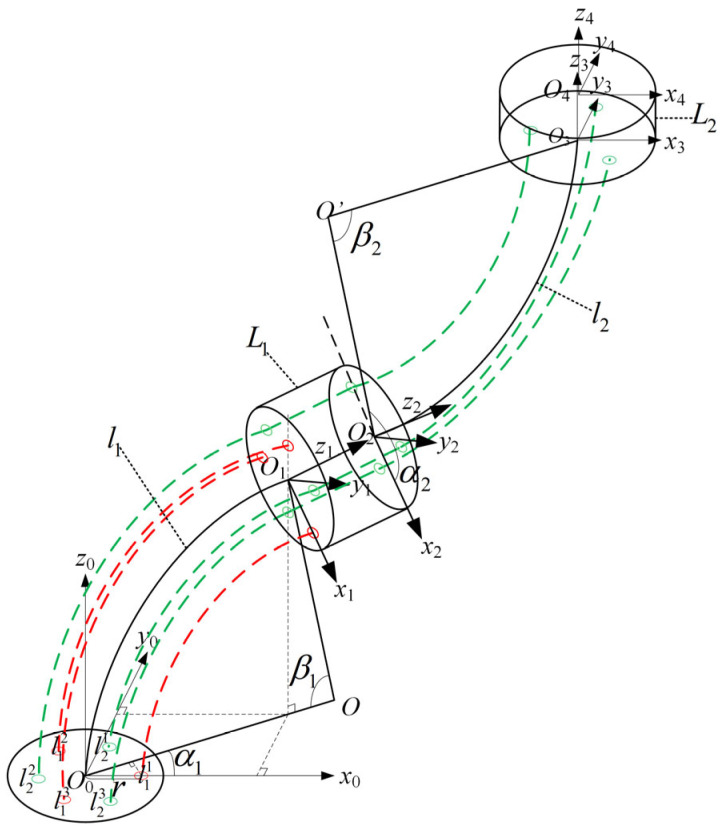
The geometric model of the two-segment continuum manipulator.

The geometric model of the two-segment continuum manipulator is shown in Figure 3. According to the designed prototype structure, the manipulator body comprises two continuous segments and two translational segments. The base coordinate system $O_{0}$ is established at the center of the base of the first continuous segment. The coordinate system $O_{1}$ is established at the center of the end of the first continuous segment. The base coordinate system $O_{2}$ of the second continuous segment is established at the end of the translational segment of the first continuous segment. The end coordinate system $O_{3}$ of the second continuous segment is established at the end of the second continuous segment, and finally, the workspace coordinate system $O_{4}$ of the robot is established at the center of the translational segment at the end of the second continuous segment. Among these, the x-axis positive direction of the base coordinate system $O_{0}$ points toward the first drive cable through-hole; the z-axis positive direction is perpendicular to the xy-plane of the base coordinate system and upward, while the y-axis positive direction follows the right-hand rule. The x-y-z-axes of all other coordinate systems align with those of the base coordinate system $O_{0}$. In the diagram, $\beta_{1}$ and $\beta_{2}$ represent the bending angles of the first and second segments relative to their respective base coordinate system planes, while $\alpha_{1}$ and $\alpha_{2}$ denote the rotation angles relative to the x-axis of their own base coordinate systems. The two-segment continuum manipulator is driven by three drive cables per segment, resulting in six through-holes arranged at 60-degree intervals counterclockwise from the x-axis in the base coordinate system $O_{0}$. These through-holes drive the motion of the two-segment continuum manipulator, where holes numbered $l_{1}^{1}\sim l_{1}^{3}$ correspond to the drive cable through-holes for the first continuous joint, $l_{2}^{1}\sim l_{2}^{3}$ correspond to those for the second continuous joint, and r denotes the distance from the base coordinate system center to the drive cable through-holes.

## 4. Inverse Kinematics Solving Based on IRRT-Connect

### 4.1. Algorithm Theory

**Figure 4:**
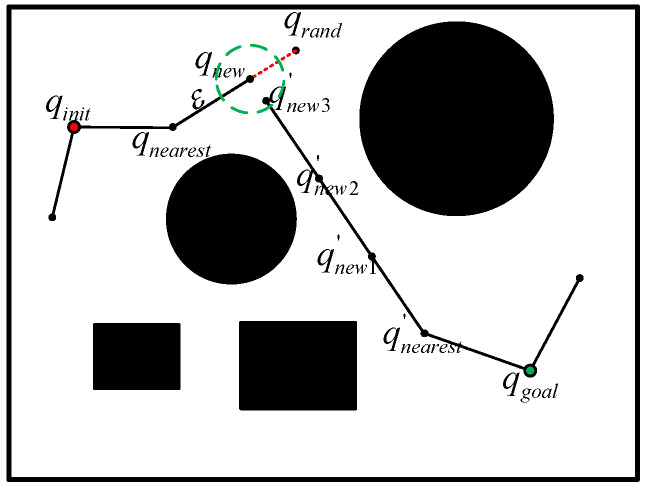
RRT-Connect schematic diagram.

Compared to the traditional RRT algorithm, RRT-Connect employs a dual-tree expansion mechanism, initiating from both the start and goal points as root nodes. Each tree extends toward the other’s direction through alternating expansion, effectively reducing planning time. Furthermore, the incorporation of heuristic steps significantly improves search speed, particularly demonstrating enhanced performance in navigating narrow passages. The principle is illustrated in Figure 4.

This section proposes an improved algorithm based on RRT-Connect for a two-segment continuum manipulator to address PTP motion planning in GIS complex cavity environments. The path planning operates in the virtual joint configuration space C of the manipulator, where $q=\left(\alpha_{1},\beta_{1},\alpha_{2},\beta_{2}\right)$ represents any configuration in C, and ε denotes the step length during RRT-Connect expansion. To address the computational inefficiencies caused by redundant nodes, the method introduces a maximized step length strategy. This is further enhanced by stepwise optimization to reduce the number of sampling nodes, along with modified pruning and collision detection mechanisms specifically adapted to the kinematic characteristics of the continuum manipulator. These refinements collectively improve memory utilization and algorithmic adaptability in complex cavity environments. The flow of the improved algorithm is shown in Table 1.

### 4.2. Step Optimization

When invoking the Connect() function for target tree $T_{g}$ expanding toward start tree $T_{i}$ extending only a fixed step length ε may result in multiple sampling points along this direction in $T_{g}$. In reality, only the farthest sampling point in that direction is required. This situation creates numerous redundant points in $T_{g}$, causing unnecessary memory waste. To resolve this issue, the single collision detection is replaced with multiple collision detections, ensuring that leaf nodes achieve the maximum feasible step length during directional expansion, thereby maximizing the step length.

When determining the new sampling point $q_{new}$, the single collision detection with step length ε is replaced by four sequential collision detections at 0.5ε, ε, 1.5ε, 2ε. This method effectively reduces the number of leaf nodes in the random expansion tree, thereby mitigating memory consumption.

(3) $$q_{new}=q_{nearest}+\varepsilon \frac{\left(q_{rand}-q_{nearest}\right)}{\left\|q_{rand}-q_{nearest}\right\|}$$

Simultaneously, the specific value of step length ε directly impacts both node quantity and solution efficiency. Therefore, a step-prior selection strategy is incorporated during the four-step greedy expansion: the difference in variation between the initial configuration $q_{init}$ and target configuration $q_{goal}$ is precomputed before algorithm iteration to further determine the value of ε. The variation difference $D_{q}$ is defined as the sum of the absolute values of each element of the vector, as follows:

(4) $$D_{q}=\sum_{i=1}^{4}\left|q_{goal}^{i}-q_{init}^{i}\right|$$

Based on the practical requirements of path planning for a two-segment continuum manipulator in GIS complex cavity environments, the step length ε is coupled with the variation value $D_{q}$. The pre-selection scheme for the step length is shown in Equation (5):

(5) $$\varepsilon =\left\{\begin{array}{c} 2\times \Delta \ldots ,D_{q}\geq Threshold_{1}\\ 1.6\times \Delta \ldots ,Threshold_{1}>D_{q}\geq Threshold_{2}\\ \Delta \ldots ,0<D_{q}<Threshold_{2} \end{array}\right.$$

where ε is the determined step length, Δ is the reference length, and $Threshold_{1}$ and $Threshold_{2}$ are the different change thresholds.

### 4.3. Pruning Strategy

The pruning strategy can be described as follows: Taking the first waypoint (i.e., $q_{\mathrm{init}}$) in the path planning sequence $Q=\left(q_{init},q_{2},\ldots ,q_{goal}\right)$ as the fixed point $q_{fixed}$, we sequentially select a point q in reverse order from $\left(q_{goal},\ldots ,q_{2}\right)$ to perform collision detection between q and $q_{fixed}$. If the actual kinematic arm configuration connecting the two waypoints q and $q_{fixed}$ does not collide with obstacles, these two points can be directly connected. The successfully connected point q is then designated as the new fixed point $q_{fixed}^{\prime }$. This process continues iteratively from $q_{goal}$ to the updated $q_{fixed}^{\prime }$ until the new fixed point can be directly connected to $q_{goal}$.

**Figure 5:**
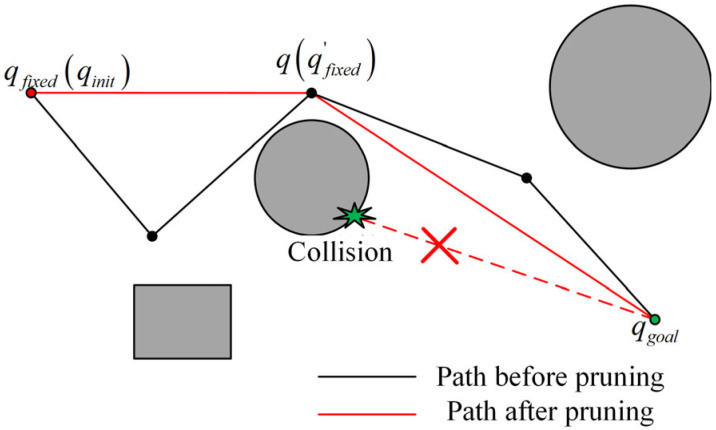
Schematic diagram of pruning strategy.

Considering the challenges in visualizing pruning strategies in high-dimensional spaces, this paper illustrates the pruning concept using a two-dimensional schematic diagram, as shown in Figure 5.

### 4.4. Collision Detection

**Figure 6:**
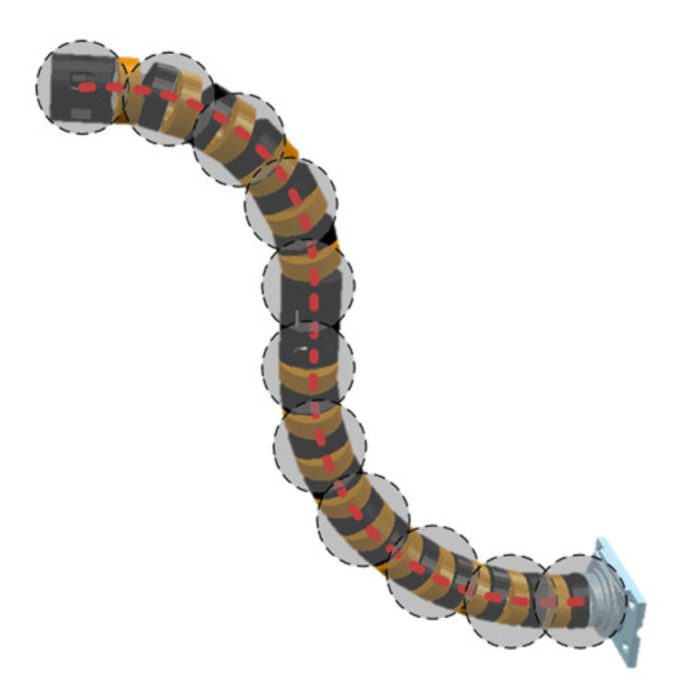
Collision model of continuum manipulator.

Since the path planning in this study operates within the virtual joint configuration space, the actual arm shape of the continuum manipulator can be derived using the forward kinematics relationship $FK_{2}\left(q\right)$ from the virtual joint space to the workspace. As shown in Figure 6, by equidistantly sampling points along the spine line of the actual arm shape based on bounding sphere positions, the spatial coordinates of all bounding spheres $\left\{P_{1},P_{2},P_{3},\ldots ,P_{i}\right\}$ are obtained.

**Figure 7:**
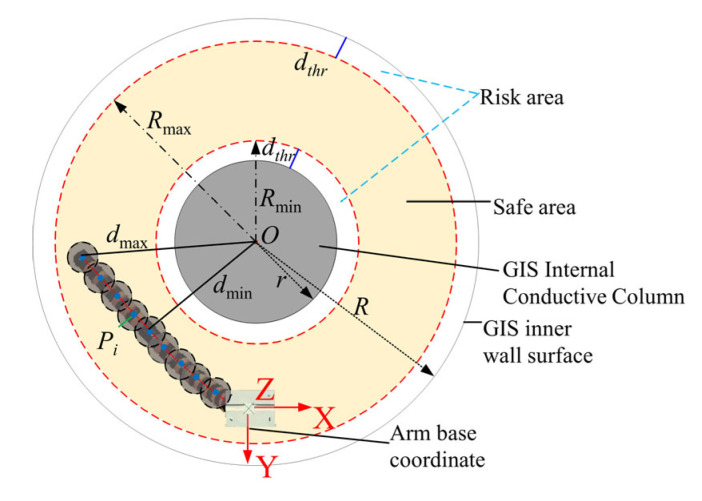
Collision detection based on dimensionality-reduction strategy.

The environmental dimension-reduction strategy transfers 3D bounding sphere collision detection to a 2D plane. This strategy implements collision detection between two-segment continuum manipulators and GIS cavity interiors, as specifically shown in Figure 7. In the figure, R,r represent the GIS metal shell radius and internal conductive column radius, respectively, $d_{thr}$ denotes the dangerous distance threshold, and $R_{\max},\;R_{\min}$ indicate the inner and outer boundary radii of the manipulator’s motion safety zone annulus.

The minimum and maximum distances between the bounding sphere model of the continuous manipulator and the O point of the GIS cavity center are as follows:

(6) $$\left\{\begin{array}{c} d_{\min}=\min\left\{d_{1},d_{2},\ldots ,d_{i}\right\}\\ d_{\max}=\max\left\{d_{1},d_{2},\ldots ,d_{i}\right\} \end{array}\right.$$

The distance $d_{i}=\left\|O^{xy}-P_{i}^{xy}\right\|$ between the bounding spheres and the GIS cavity center must ensure whether both $d_{\min}$ and $d_{\max}$ lie within the annular safety zone formed by $R_{\min}$ to $R_{\max}$.

### 4.5. Trajectory Planning Based on Driving Space

The IRRT-Connect algorithm obtains point-to-point path planning results for a two-segment continuum manipulator in GIS complex environments. Based on these results, further trajectory planning is required, with the trajectory planning outcomes directly serving as the underlying input for controlling the manipulator’s motion.

Driving-line trajectory planning involves two steps: (a) quintic polynomial interpolation within the virtual joint space; (b) deriving driving-line variation trajectories from the virtual joint-space planning results.

The virtual joint angle position change function is expressed as follows:

(7) $$q_{i}\left(t\right)=a_{i0}+a_{i1}t+a_{i2}t^{2}+a_{i3}t^{3}+a_{i4}t^{4}+a_{i5}t^{5}\;{\dot{q}}_{i}\left(t\right)=a_{i1}+2a_{i2}t+3a_{i3}t^{2}+4a_{i4}t^{3}+5a_{i5}t^{4}a\;{\ddot{q}}_{i}\left(t\right)=2a_{i2}+6a_{i3}t+12a_{i4}t^{2}+20a_{i5}t^{3}$$

By constraining the virtual joint angular position, velocity, and acceleration corresponding to the starting point at the initial time $t_{0}$ and the target point at the end time $t_{f}$, the polynomial coefficients can be obtained as follows:

(8) $$\left\{\begin{array}{c} a_{i0}=q_{i0}\\ a_{i1}={\dot{q}}_{i0}\\ a_{i2}=\frac{{\ddot{q}}_{i0}}{2}\\ a_{i3}=\frac{\left({\ddot{q}}_{if}-3{\ddot{q}}_{i0}\right){\left(t_{f}-t_{0}\right)}^{2}-\left(8{\dot{q}}_{if}+12{\dot{q}}_{i0}\right)\left(t_{f}-t_{0}\right)+20\left(q_{if}-q_{i0}\right)}{2{\left(t_{f}-t_{0}\right)}^{3}}\\ a_{i4}=\frac{\left(3{\ddot{q}}_{i0}-2{\ddot{q}}_{if}\right){\left(t_{f}-t_{0}\right)}^{2}+\left(14{\dot{q}}_{if}+16{\dot{q}}_{i0}\right)\left(t_{f}-t_{0}\right)+30\left(q_{if}-q_{i0}\right)}{2{\left(t_{f}-t_{0}\right)}^{4}}\\ a_{i5}=\frac{\left({\ddot{q}}_{if}-{\ddot{q}}_{i0}\right){\left(t_{f}-t_{0}\right)}^{2}-\left(6{\dot{q}}_{if}+6{\dot{q}}_{i0}\right)\left(t_{f}-t_{0}\right)+12\left(q_{if}-q_{i0}\right)}{2{\left(t_{f}-t_{0}\right)}^{5}} \end{array}\right.$$

## 5. Simulation Results and Analysis

The prototype model is set with the parameters of the continuum robot. The length of joint segment 1 is $l_{1}=173$ mm, the length of translation segment 1 is $L_{1}=18$ mm, the length of joint segment 2 is $l_{2}=101$ mm, and the length of translation segment 2 is $L_{2}=12$ mm. The distance r from the center of the base coordinate system to the center of the drive cable through-hole is 7.25 mm. The performance parameters of the computer used in the simulation are as follows: the CPU is Intel^®^ Core^TM^ i7-10700, the memory (RAM) is 16 GB, the graphics card is NVIDIA GTX 1660 SUPER (From ASUS, Suzhou, China), and the MATLAB version is 2018a.

### 5.1. IRRT-Connect Algorithm Simulation

In the simulation, the data with a large difference between the initial and target configuration changes are selected as example ➀, and the data with a small difference are selected as example ➁.

**Figure 8:**
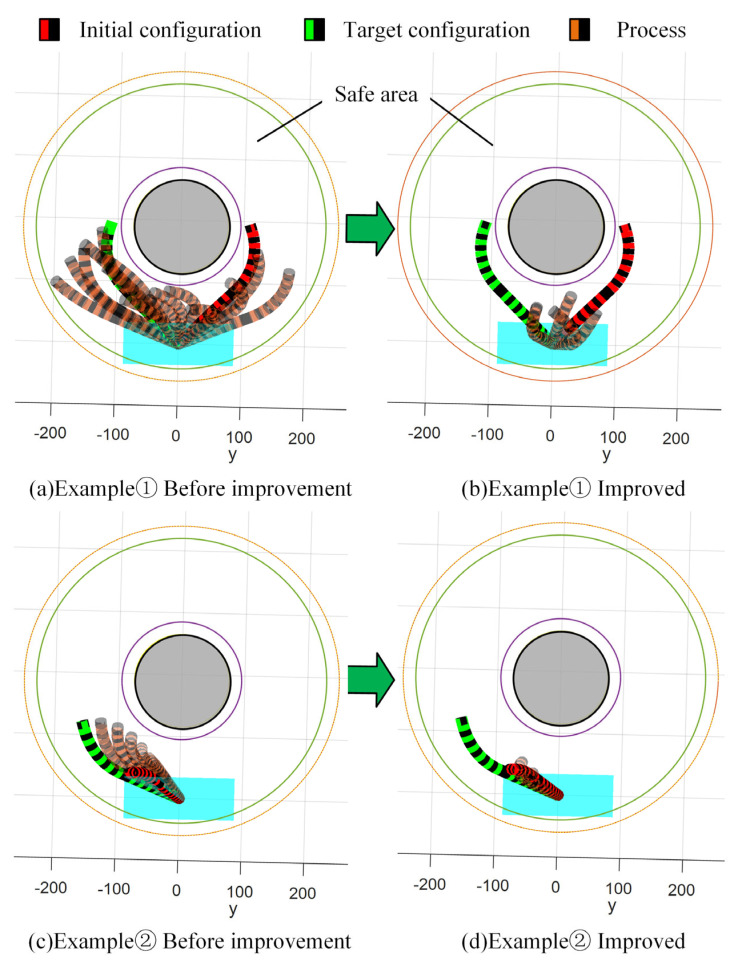
Comparison of planning results before and after RRT-connect algorithm improvement.

The specific data of simulation example ➀: Posture from beginning to end (−195.35, 109.31, 110.14, 1.89, −0.78, −1.57)→(−195.35, −109.31, 110.14, −1.89, −0.78, 1.57), and corresponding start and end virtual joint variables (1.57, 2.36, 1.10, 3.93)→(1.57, 3.93, 1.10, 2.36). The specific data of simulation example ➁: Posture from beginning to end (−39.56, −77.83, 287.64, 0.06, 0.02, 0.33)→(−121.57, −156.58, 179.71, −1.23, −0.67, 1.45), corresponding start and end virtual joint variables (0.47, 4.08, 0.31, 0.16)→(1.10, 4.32, 0.94, 2.98). In the GIS cavity simulation environment, the basic RRT-connect algorithm and the IRRT-connect algorithm are used to carry out 100 planning experiments on two groups of instances, respectively, and the specific data are shown in Table 2. The last experimental planning results are shown in Figure 8. The results obtained from 100 planning experiments are as follows.

### 5.2. Drive Trajectory Smoothing Test

**Figure 9:**
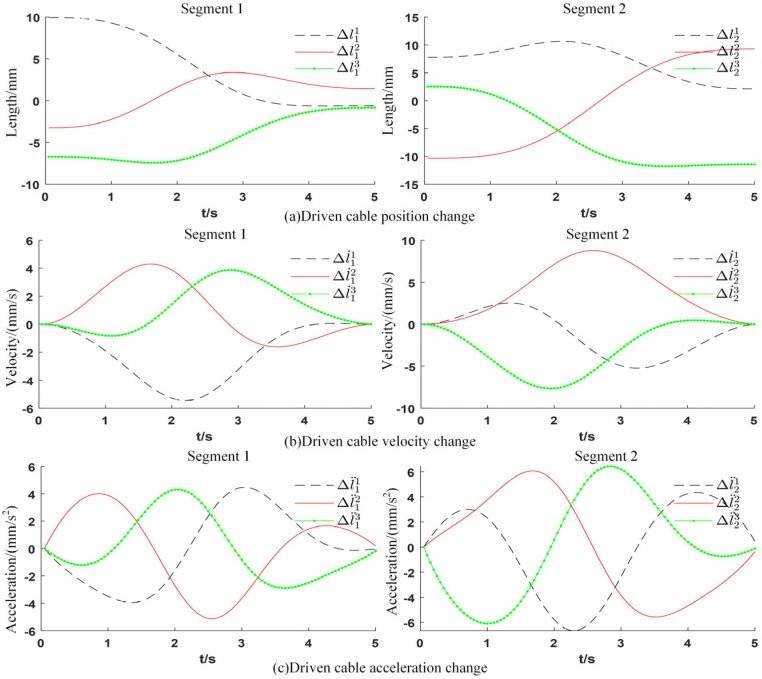
Polynomial trajectory planning.

To validate the practical effectiveness of applying the quintic polynomial trajectory planning method to a two-segment continuum manipulator, a point-to-point motion case is selected for simulation testing, and the example parameters are set as follows. Virtual joint configuration $q_{init}=\left(0.2,2,1.5,2.5\right)$ is the starting point of polynomial trajectory planning, $q_{goal}=\left(1.4,0.2,0.15,1.2\right)$ is the target point, and the speed constraint ${\dot{q}}_{init}=0,\;{\dot{q}}_{goal}=0$ and acceleration constraint ${\ddot{q}}_{init}=0,\;{\ddot{q}}_{goal}=0$ are set at the same time. The time from the starting point to the target point is 5 s, so $t_{0}=0,\;t_{f}=5s$. The results are shown in Figure 9.

### 5.3. Co-Simulation of MATLAB and CopeliaSim

**Figure 10:**
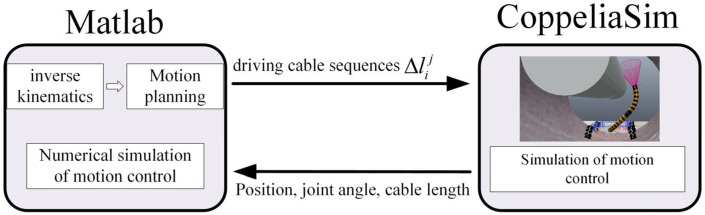
Motion control simulation program.

**Figure 11:**
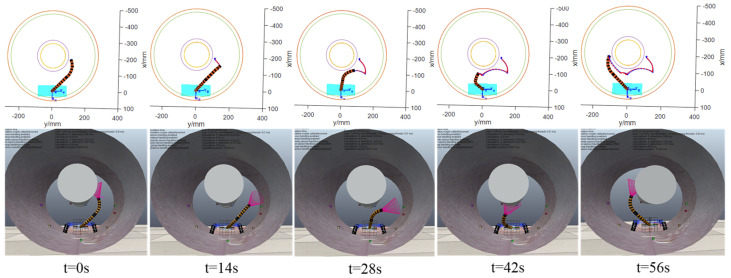
Numerical simulation (**top**) and virtual simulation (**bottom**) motion control timing diagrams.

**Figure 12:**
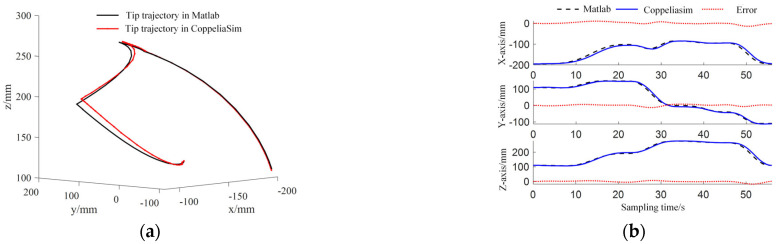
End trajectory comparison. (**a**) Tip trajectory comparison; (**b**) comparison of changes in spatial coordinates.

The motion planning process from initial virtual joint variables (1.57, 2.36, 1.10, 3.93) to target virtual joint variables (1.57, 3.93, 1.10, 2.36) is accomplished using the improved RRT-Connect algorithm and quintic polynomial interpolation. As shown in Figure 10, the planned results (six driving cable sequences $\Delta l_{i}^{j}$) are transmitted in real time to the CoppeliaSim virtual simulation platform to control the two-segment continuum manipulator’s virtual model. To compare virtual simulation motion control effects, a 550 KV GIS cavity and two-segment continuum manipulator numerical simulation model are implemented in MATLAB. During motion control simulations, MATLAB numerical simulations and CoppeliaSim virtual model simulations are synchronized. As shown in Figure 11, the timing comparison between its bitwise numerical and virtual simulation motion control. The end trajectory comparison is shown in Figure 12

## 6. Experiment and Result Analysis

### 6.1. Experiment Preparation and Process

**Figure 13:**
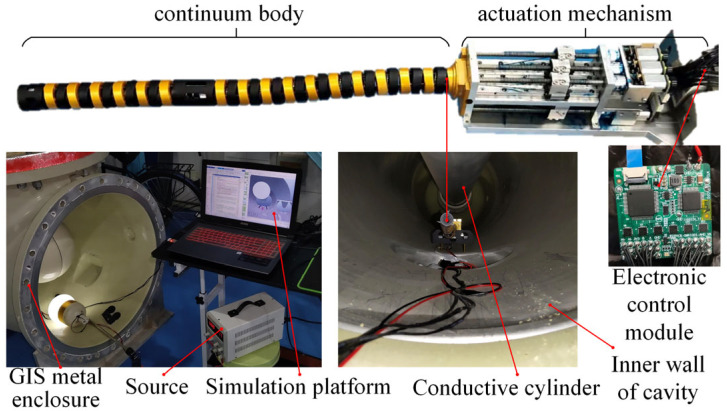
Prototype and experimental environment.

To verify the effectiveness of the proposed obstacle-avoidance planning algorithm, a GIS experimental environment as shown in Figure 13 is built. The experimental environment includes a two-stage continuous manipulator prototype, an electric control system, and a real 500 kV GIS tank. The electric control system uses STM32 (From STMicroelectronics, Shenzhen, China) embedded processor to drive and control six DC motors connected to the driving cables.

The prototype of the two-stage continuum manipulator is composed of a continuum body, a driving mechanism, and an electronic control module. The continuum body is made of nylon material by 3D printing. The electronic control module uses an STM32 chip as the underlying microcontroller to drive the micro DC motor which can control the expansion and contraction of the drive line. The experimental environment is 550 kV GIS equipment provided by State Grid Corporation.

**Figure 14:**
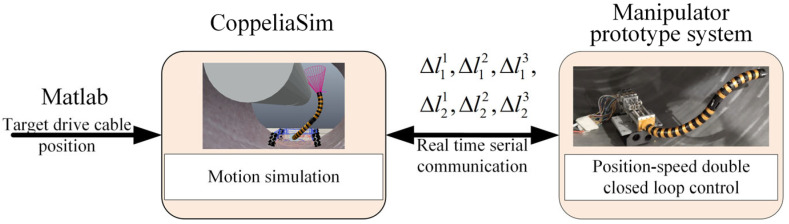
Prototype experimental control block diagram.

In the GIS cavity application scenario, the schematic diagram of the point-to-point (PTP) motion control experiment for a two-segment continuum manipulator is shown in Figure 14. The CoppeliaSim platform achieves real-time communication with the underlying microcontroller STM32 (From STMicroelectronics, Shenzhen, China) via serial port. During synchronized simulations between MATLAB and CoppeliaSim, the target driving cable sequence $\left(\Delta l_{1}^{1},\;\Delta l_{1}^{2},\;\Delta l_{1}^{3},\;\Delta l_{2}^{1},\;\Delta l_{2}^{2},\;\Delta l_{2}^{3}\right)$ from CoppeliaSim is transmitted in real time to the STM32 microcontroller through the serial interface, enabling synchronous planning and control among all three systems.

The drive commands sent through the serial port reach the underlying microcontroller of the robot with a delay controlled within 35 ms, which is less than the motor execution frequency of 50 ms.

### 6.2. Analysis of Experimental Results

**Figure 15:**
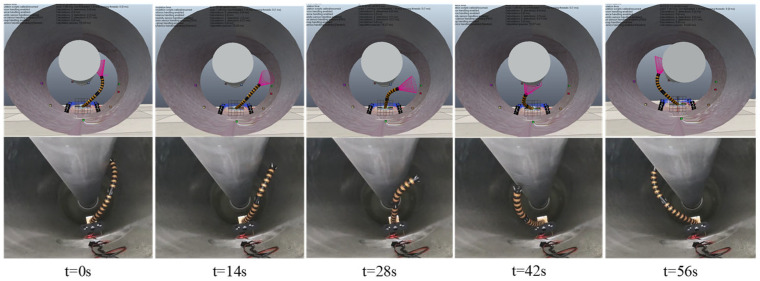
Comparison of motion control between virtual simulation (**top**) and physical experiments (**bottom**).

**Figure 16:**
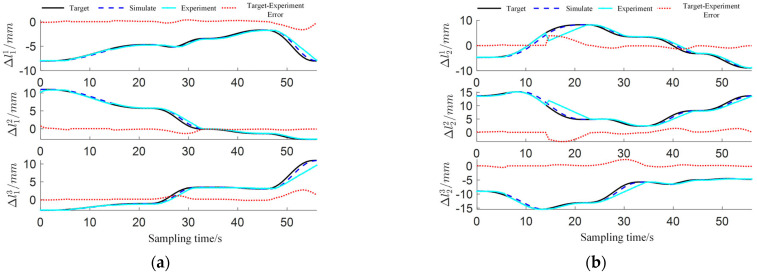
Driven cable comparison: (**a**) segment 1 driven cable comparison; (**b**) segment 2 driven cable comparison.

As shown in Figure 15, the comparative timing diagrams of virtual simulation and physical experiment motion control reveal that the physical continuum manipulator and simulation model exhibit broadly similar motion processes during PTP operations. Due to the narrow and complex internal structure of GIS equipment, existing measurement techniques cannot reliably capture the end-effector trajectory or virtual joint variables during point-to-point motion of the two-segment continuum manipulator. Experimental data acquisition is limited to real-time driving cable positional changes obtained from motor encoder feedback. Comparative analyses between target, simulated, and experimental positional values of the manipulator’s six driving cables are shown in Figure 16a,b. The diagrams indicate intermittent positional errors during experimental phases, potentially attributable to mechanical inaccuracies in the continuum body prototype causing motor stalling or inherent encoder errors in DC motors. Despite observed deviations, the results demonstrate the feasibility of deploying two-segment continuum manipulators for GIS cavity inspection and maintenance tasks, confirming that the proposed motion planning methodology achieves effective PTP motion planning and control in complex GIS cavity environments.

## 7. Conclusions

To address the collision-avoidance shape planning challenges in key-area fixed-point inspection tasks for GIS equipment maintenance, this study proposes a collision-avoidance planning method for continuum manipulators based on an improved RRT-Connect algorithm. The method transforms Cartesian-space collision avoidance into virtual joint-space obstacle evasion. First, a joint-space obstacle map is established, and the improved RRT-Connect algorithm enhances computational efficiency by reducing node quantity and expansion rate. Furthermore, polynomial-driven spatial trajectory planning calculates smooth driving-line variations to control the dual-segment continuum manipulator’s end-effector toward target positions. Simulation and experimental validation on a two-segment continuum manipulator demonstrates the proposed C-space collision-avoidance shape planning method achieves effective and rapid performance, laying the groundwork for real-time manipulator shape planning. For practical implementation, future work will incorporate end-effector motion constraints to extend from point-to-point obstacle-avoidance motion planning to continuous path motion planning, and to achieve wide constraints on end position without significantly increasing the planning time, thus further improving the operational efficiency of the continuum manipulator.

## Figures and Tables

**Table 1:** Algorithm pseudocode.

RRT-Connect Planner for Continuum Manipulator (*x_init_*, *x_goal_*, *Map*)	
1	*T*_*a*_.init(*x*_*init*_);*T_b_*.init(*x*_*goal*_);
2	for *k* = 1 to *K* do
3	*x*_*rand*_←*SampleNode*(*Step Optimization*):
4	if not(*Extend*(*T*_*a*_, *x*_*rand*_)) = Trapped then
5	if (*Connect*(*T*_*b*_, *x*_*new*_)) = Reached then
6	Return PATH(*T*_*a*_, *T*_*b*_);
7	end if
8	end if
9	$\mathrm{if}\;\left|T_{a}\right|>\left|T_{b}\right|$ then
10	SwapTrees(*T*_*a*_, *T*_*b*_);
11	end if
12	end for
13	*PathPruning*()
14	Return Failure

**Table 2:** Comparison of RRT-connect algorithm improvement efficiency.

**Example**	**Algorithm**	**Average Number of Iterations**	**Average Number of Leaf Nodes**	**Average Number of Path Nodes**	**Average Number of Path Nodes**
➀	RRT-Connect	150	46	19	0.017 s
	IRRT-Connect	48	12	8	0.011 s
➁	RRT-Connect	2	8	8	0.002 s
	IRRT-Connect	5	5	4	0.002 s

## Data Availability

Data are contained within this article. Further inquiries can be directed to the corresponding author.

## Abbreviations

The following abbreviations are used in this manuscript:

IRRT	Improved rapidly exploring random tree
GIS	gas-insulated switchgear
C-space	configuration space
PRM	probabilistic roadmap
